# ELBW survivors in early adulthood have higher hepatic, pancreatic and subcutaneous fat

**DOI:** 10.1038/srep31560

**Published:** 2016-08-17

**Authors:** Justin D. Crane, Samuel A. Yellin, Frank J. Ong, Nina P. Singh, Norman Konyer, Michael D. Noseworthy, Louis A. Schmidt, Saroj Saigal, Katherine M. Morrison

**Affiliations:** 1 Department of Pediatrics, McMaster University, Hamilton, ON, Canada; 2 Department of Biology, Northeastern University, Boston, MA, USA; 3 Department of Radiology, McMaster University Medical Center, Hamilton, ON, Canada; 4 Imaging Research Centre, St. Joseph’s Healthcare, Hamilton, ON, Canada; 5 Department of Electrical and Computer Engineering, McMaster University, Hamilton, ON, Canada; 6 Department of Psychology, Neuroscience and Behaviour, McMaster University, Hamilton, ON, Canada

## Abstract

Premature birth in conjunction with extremely low birth weight (<1 kg, ELBW) is associated with insulin resistance and increased cardiometabolic health risk compared to birth at full term with normal birth weight (NBW). However, little is known regarding the biologic mediators of these effects. Abdominal and ectopic lipid accumulation is linked to insulin resistance and metabolic dysfunction, yet whether ELBW survivors are predisposed to aberrant lipid deposition in adulthood is unknown. We used magnetic resonance imaging in a cohort of 16 NBW and 29 ELBW participants to determine if ELBW survivors have differences in pancreatic, hepatic, subcutaneous and visceral fat distribution compared to NBW participants. ELBW individuals had a higher proportion of liver and pancreatic fat compared to NBW subjects (*P* < 0.05). Abdominal subcutaneous fat, but not visceral fat, area was higher in ELBW survivors compared to NBW individuals. In multivariate analyses, tissue fat measures were most highly related to BMI and sex, but not preterm birth. This work highlights that fat deposition is enhanced in adults born preterm and suggests that ectopic fat accretion driven by their relatively greater adiposity may contribute to the higher rates of metabolic dysfunction seen in ELBW survivors.

Infants born prior to 37 weeks of gestation, or premature infants, represent approximately 10% of all births in developed countries^1^. Preterm babies miss a critical period of growth and development in utero and often require additional post-natal and pediatric medical care. The first preterm individuals treated in modern neonatal intensive care environments with routine use of monitoring and life-support systems are now reaching their fourth decade of life. Extreme premature birth (categorized by birth weights under 1500 g) is associated with a greater risk of metabolic and cardiovascular dysfunction later in life through adulthood^2^,^3^,^4^,^5^. Adverse health outcomes include high blood pressure, greater insulin resistance and a higher prevalence of dysglycemia. Emerging research suggests that the greater cardiometabolic health risk seen in adults born preterm is due to inappropriate fetal development of metabolic tissues, including muscle and fat. When not formed appropriately, the insufficient storage and oxidation of glucose, fatty acids and lipids by metabolic tissues leads to hyperglycemia, hyperlipidemia and insulin resistance. A hallmark of incomplete fat metabolism is the excessive deposition of ectopic fat and its infiltration within and around abdominal organs, a condition that has emerged as a leading predictor of cardiovascular risk and dysglycemia^6^. However, whether adults born premature are predisposed to alterations in abdominal or organ fat accumulation remains poorly understood.

Adipose tissue development in utero begins during the second trimester where it is initially deposited in the head and neck, followed by the trunk and upper and lower limbs^7^. By the beginning of the third trimester, subcutaneous adipose tissue is present in all of the typical depots seen later in life and expands rapidly compared to the first and second trimesters^7^. Therefore, premature birth in the late second and early third trimesters may interfere with a critical period of adipose tissue expansion^8^,^9^. Born leaner and with lower adiposity, premature infants often experience an accelerated period of “catch-up” post-natal adipose tissue growth that supersedes the relative rate of muscle growth^10^. Interestingly, this growth pattern is similar to that seen in individuals born at full term with low birth weight and is thought to underlie observations that low birth weight individuals have more abdominal^11^,^12^ or truncal^13^,^14^ adipose tissue and increased metabolic health risk^15^,^16^,^17^ as adults.

In adults, accumulated abdominal fat, particularly within the visceral compartment, is associated with dysglycemia, dyslipidemia and insulin resistance^18^,^19^ and is a major cardiovascular risk factor^20^. Visceral fat has several characteristics that may be especially harmful to other organs including greater inflammation and excess free fatty acid efflux leading to spill over into other tissues^21^. Similarly, evidence suggests an increased proportion of fat within major metabolic organs such as the liver and pancreas may be partially responsible for the metabolic dysfunction that leads to dysglycemia and diabetes^22^,^23^. However, whether extremely low birth weight and preterm birth are associated with alterations in abdominal, hepatic and pancreatic fat accumulation is not clear. As ELBW birth has been connected with higher adiposity and a greater prevalence of dysglycemia^24^ and prematurity has been linked to type 2 diabetes^25^,^26^, the characterization of different abdominal adipose depots may provide insight into the pathophysiological consequences of premature birth.

Babies born with extreme prematurity require oxygen therapy due to immature lung development. While life saving, high oxygen exposure in the premature infant has been linked to the development of retinopathy of prematurity (ROP) leading to blindness or vision problems^27^. As pancreatic tissue is similarly sensitive to oxidative stress^28^,^29^, and given that ROP was associated with 2 hour blood glucose in our full cohort^24^, we were interested in further examining the potential relationship of ROP to pancreatic fat content and volume.

Our primary objectives were to compare fat distribution and hepatic and pancreatic volume and fat content in young adults born ELBW compared to NBW controls. We also wanted to examine the influence of current body size, birthweight group and the presence of ROP on organ volume and fat content. We hypothesized that adults born extremely premature (ELBW) would have higher hepatic and pancreatic fat fractions and a greater area of subcutaneous and visceral fat and that those with ROP would have higher pancreatic fat and lower pancreatic volume.

## Results

The physical characteristics of the subjects at the time of the MRI scan are presented in Table 1. As expected, ELBW survivors were of shorter stature than NBW individuals, yet were of similar body weight. While there was a tendency for ELBW survivors to have a greater body mass index (BMI) and to be classified as obese (BMI > 30 kg/m^2^), these did not reach statistical significance.

**Figure 1:**
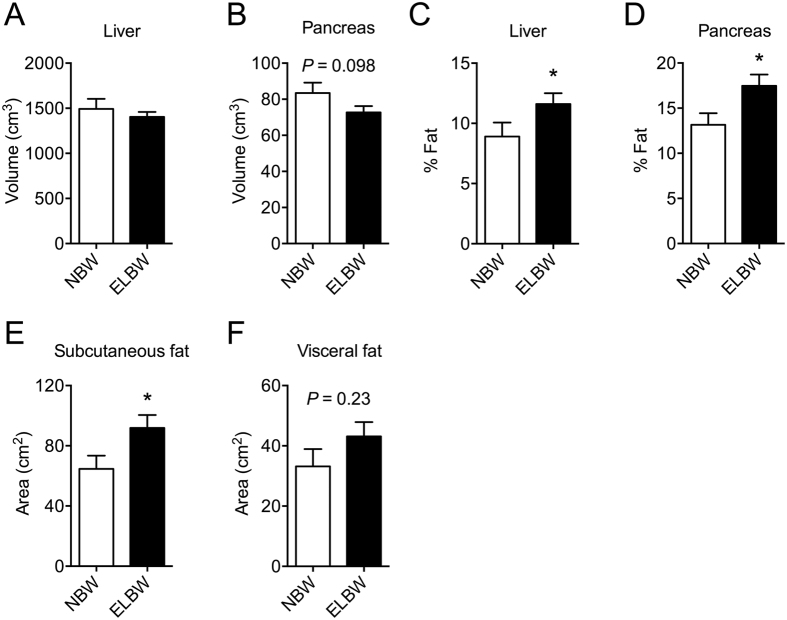
ELBW individuals have dysregulated ectopic fat deposition. (**A**) Liver and (**B**) pancreatic total organ volume and (**C**) liver and (**D**) pancreatic fat fraction in NBW and ELBW subjects as assessed by MRI. (**E**) Subcutaneous and (**F**) visceral fat area at 5 cm above L4/5 interface. *Indicates a significant difference (*P* < 0.05) from NBW group. Data are mean ± SEM.

NBW and ELBW participants had comparable liver and pancreas volumes (Fig. 1A,B). Similar results were obtained when organ volumes were adjusted for height (liver volume, *P* = 0.76; pancreas volume, *P* = 0.50). As the proportion of fat within the liver and pancreas has been inversely associated with metabolic health, we assessed the fat fraction in these organs. Consistent with previous studies^30^, liver and pancreatic fat fractions were strongly correlated. Similarly, subcutaneous and visceral fat volumes were also tightly correlated (Supplementary Table S1). ELBW individuals had higher hepatic and pancreatic fat fractions compared to NBW (Fig. 1C,D). ELBW survivors also exhibited greater subcutaneous, but not visceral adipose tissue area compared to NBW subjects (Fig. 1E,F). Hepatic and pancreatic fat fraction and subcutaneous and visceral fat area did not differ between ELBW individuals born small for gestational age compared to those born appropriate for gestational age (data not shown).

To determine if ELBW birth had an independent influence on organ fat, or if the influence was through current body size, we conducted a multivariate analysis including group (ELBW vs NBW), sex and BMI. Sex and BMI were the strongest predictors of all fat depots (Table 2) and ELBW birth had no independent relationship to pancreatic, hepatic, visceral or subcutaneous fat. This suggests that the fat distribution differences seen between groups are a result of a tendency to have a higher BMI and not to birthweight group.

We also sought to determine if the common ELBW condition ROP was related to organ volume. We compared hepatic and pancreatic organ volumes of ELBW survivors with diagnosed ROP (only those with stage I-III were identified, *n* = 6) relative to those with no known ROP (*n* = 19). When comparing the MRI organ measurements, subjects with ROP had similar liver volumes compared to subjects without ROP (1390 ± 76 vs.1425 ± 75 cm^3^, respectively, mean ± SEM; *P* = 0.80). However, ROP-diagnosed individuals had smaller pancreas volumes than those without ROP (60.7 ± 8.6 vs. 79.6 ± 3.8 cm^3^, respectively, mean ± SEM; *P* = 0.03), suggesting a possible relationship between pancreatic development and hyperoxia in ELBW survivors. Liver and pancreatic fat were comparable between subjects with and without ROP (*P* = 0.75 and *P* = 0.32 respectively). As those ELBW with ROP also had a more challenging neonatal course as evidenced by the longer duration of respiratory support required (54 ± 15 vs. 17 ± 5 days, respectively, mean ± SEM; *P* = 0.008) and the later age at time of discharge (103 ± 17 vs. 57 ± 6 days, respectively, mean ± SEM; *P* = 0.004), we cannot conclude that hyperoxia has caused the smaller pancreatic volume. It does suggest however, that perhaps ROP may be a marker of future glycemic disturbances.

Cardiometabolic health and body composition were first measured in the whole available cohort on average 2 years prior to the MRI studies (descriptive data are in Supplementary Table S2). As hepatic and abdominal fat have been strongly linked with increased cardiometabolic risk, we wanted to determine if the greater subcutaneous, hepatic and pancreatic fat in the ELBW group were associated with the previously measured cardiometabolic risk factors. Cardiometabolic variables are often reported to be stable in control groups over a period of 2 years^31^,^32^,^33^, justifying our comparison of measurements collected over this time span. Hepatic and pancreatic fat fraction were directly related to systolic blood pressure (liver fat fraction: r = 0.33; pancreatic fat fraction: r = 0.51, *P* < 0.05), triglycerides (liver fat fraction: r = 0.54; pancreatic fat fraction: r = 0.55, *P* < 0.05) and HOMA-IR (liver fat fraction: r = 0.56; pancreatic fat fraction: r = 0.37, *P* < 0.05) and inversely related to HDL-cholesterol (liver fat fraction: r = −0.52; pancreatic fat fraction: r = −0.49; *P* < 0.05). Visceral fat area and pancreatic fat fraction were directly related to fasting plasma glucose (visceral fat area: r = 0.44; pancreatic fat fraction: r = 0.31, *P* < 0.05) and insulin (visceral fat area: r = 0.40; pancreatic fat fraction: r = 0.36, *P* < 0.05).

## Discussion

Adult survivors of ELBW birth have higher hepatic and pancreatic fat fractions and higher subcutaneous fat area compared to NBW controls. These results confirm and extend prior reports that differential adipose tissue accumulation occurs in adults born with extreme prematurity and may underlie the reported greater cardiometabolic risk in these individuals. Consistent with this premise, we also identified a moderate direct relationship between liver fat and previously determined systolic blood pressure and fasting triglyceride levels suggesting linkages to increased cardiometabolic risk even at these relatively low levels of liver fat.

Only one prior study has investigated fat distribution and ectopic fat in adults born premature. This study assessed younger subjects (~24 years old; *n* = 25 term and *n* = 23 preterm) and found greater hepatic, intramuscular and abdominal fat content in adults born preterm (mean gestational age of 29 weeks) compared to normal birthweight term controls^5^. Although similar levels of liver and pancreatic fat as seen in our cohort have been connected to dysglycemia^34^,^35^, the average relative fat content in the liver of our ELBW individuals (~12%) is lower than that observed in obese populations^36^ or in those with non-alcoholic fatty liver disease^37^.

No prior investigations have assessed pancreatic fat in ELBW survivors. Pancreatic fat, like liver fat, has been strongly associated with obesity, insulin resistance and dysglycemia^34^,^36^,^38^,^39^. Given the sensitivity of the pancreas to oxidative stress, we investigated the relationship of ROP, an oxygen mediated condition that develops in premature infants^40^, to pancreatic volume. Although a preliminary finding given the small sample size, we identified lower pancreatic volume in those ELBW individuals with evidence of ROP compared to those who did not. Confirmation of this finding and the relationship of ROP to dysglycemia in adults born premature should be pursued.

Premature born adults have, in a previous study, demonstrated greater lipolysis^41^, a hallmark of visceral white adipose tissue dysfunction, which often is associated with higher levels of organ fat. Additionally, a prior study found greater visceral fat volume in adults born preterm^5^. Interestingly, we did not see elevated visceral fat content in ~30 year old ELBW subjects, despite elevated hepatic, pancreatic, and subcutaneous fat deposition.

Only sex and BMI, but not premature birth, were independently associated with ectopic fat levels in multivariate analysis, suggesting that the greater relative adiposity of ELBW subjects is the driving force behind fat accrual in these depots. This is an important finding as it suggests that prevention of excess adiposity may alleviate the increased risk of ectopic fat accumulation in those born premature. It remains to be seen whether the greater fat accumulation in ELBW survivors is modifiable by dietary, lifestyle or pharmacologic interventions.

Because extremely premature babies experience a critical period of adipose and skeletal muscle tissue development outside the womb, it is plausible that this may deter normal development of these tissues across the lifespan. Future work is required to discern the primary developmental determinants of fat accretion and fat distribution as well as a detailed growth trajectory of tissue fat from birth. In the context of our findings, there are several possible consequences of interrupted adipose tissue development in ELBW survivors that could underlie their increased cardiometabolic risk: First, dysregulated tissue remodeling and immune cell infiltration causing greater low-grade inflammation, a hallmark of insulin resistance and metabolic syndrome and second, lower functioning adipose and muscle progenitor cells due to extreme preterm birth, impairing their number, renewal or recruitment into mature tissue later in the life course, thus impairing organ function. Therefore, an analysis of circulating inflammatory markers from blood or the measurement of resident stem cells in adipose or muscle tissue samples from these individuals would help clarify the cellular alterations caused by extreme preterm birth.

There are several potential limitations in the current study. Due to financial and logistical limitations, we were only able to examine a portion of the available ELBW cohort. Based on a previous study, we estimated that a cohort of 45 individuals would provide sufficient power to identify a difference in hepatic fat. We sought to randomly select the 45 individuals from the larger study cohort – but acknowledge that as all individuals contacted did not agree to participate, we may have introduced some bias into our selection. A second limitation is that we did not discern whether the accrual of organ fat was due to alterations in basal metabolic rate. A large portion of whole body energy expenditure is driven by skeletal muscle, but emerging evidence has highlighted a potential role of brown adipose tissue as an energetic, anti-obesity tissue in adults^42^. Since ELBW survivors have fetal and postnatal stress during a critical period of adipose tissue development there may be underlying brown adipose tissue dysfunction that persists into adulthood. Thus, reduced thermogenic brown adipose tissue metabolism could partially explain the greater relative adiposity of the ELBW group. Future work should assess the contribution of brown and white adipose tissue as well as skeletal muscle to whole body energy metabolism in ELBW survivors. There was a time delay between the initial visit when glycemia, body composition and blood pressure were assessed and when the MRI scans were performed. Thus, some of the cardiometabolic characteristics could have changed by the time of the MRI scan. Thus, the relationship between cardiometabolic health and fat distribution may be stronger than demonstrated in our findings. However, only subtle changes in BMI occurred between these two visits (see Methods section), and we feel that it is unlikely these alterations strongly influenced the relationships between anthropometric characteristics and organ fat.

## Methods

Subjects were recruited as a subset of a larger cohort of individuals that have been followed since birth (1977–1982) in the Greater Hamilton, Ontario region. This cohort consists of ELBW survivors recruited at birth (weight < 1,000 grams) in a population representative manner and NBW subjects group-matched for sex, age, and socioeconomic status who were enrolled when both groups were 8 years old^43^. The subjects invited for an MRI visit were randomly selected from a list of participants who had metabolic studies and a dual-energy X-ray absorptiometry (DXA) scan done approximately 2 years prior to this study and who lived within one hour of the study site. A total of 94 individuals were invited to the MRI visit prior to completion of planned study enrollment. Of these, 19 were ineligible as they could not have an MRI done (presence of a foreign body, pregnancy, body weight exceeding 300 pounds, claustrophobia, vertigo), 19 potential participants declined to participate (lack of interest, did not have time to participate, or no specific reason was given), 8 could not be contacted and 2 cancelled their scheduled visit. Thus, 46 were enrolled and completed the study. All testing was performed in accordance with the approved guidelines. All subjects gave informed consent and the Hamilton Health Sciences Research Ethics Board approved all procedures.

After consenting, the single visit included completion of questionnaires, anthropometric measures (height, weight, BMI calculation) and abdominal MRI scanning. The abdominal MRI scans were acquired using a 3 Tesla whole-body MRI scanner (Discovery 750, GE Healthcare, Waukesha, WI, USA) with a 32-channel torso array coil (NeoCoil, Pewaukee, WI, USA). A 3D gradient-echo based pulse sequence with chemical-shift based water-fat separation was used: LAVA-FLEX, axial plane, repetition time (TR) 3.75 ms, echo time (TE) 1.67 ms, field of view (FOV) 400 mm, in-plane resolution 1.56 mm, slice thickness 4 mm, number of slices 56, flip angle 12 degrees, acceleration factor 2. Total scan time was approximately 20 seconds, and was done during a breath-hold. This pulse sequence generates four distinct image contrasts: water-only, fat-only, in-phase and out-of-phase images. Two locations were scanned: the first included the liver and pancreas, the second was centered at L4/L5.

Pancreatic and hepatic fat fraction were analyzed using Slice-O-Matic software by segmenting each organ volume and then co-registering the water-only and fat-only LAVA-FLEX images according to the equation: fat fraction = fat/(water + fat). Each image series was manually segmented by a single evaluator who was blinded to the group. The accuracy of the segmentation was confirmed by an experienced radiologist (N. Singh). Visceral and subcutaneous fat area were determined using the fat-only IDEAL image at a single slice 5 cm above the L4/5 interface. This method of quantification was chosen because prior reports have indicated that single slice measurements of visceral fat at L4/5 + 5 cm have a very good correlation with total visceral abdominal fat volume in both males and females^44^.

Measurement of waist circumference, blood pressure, blood sampling and the DXA scan for body composition occurred at a separate visit preceding the MRI visit by approximately 2 years and have not been previously published (2.2 ± 0.1 year difference between visits, mean ± SEM, outcomes in Table 2). There was a small reduction in BMI between this visit and the MRI scan in the NBW group (∆BMI NBW: −0.76 ± 0.31 kg/m^2^, *P* = 0.03; ∆BMI ELBW: 0.54 ± 0.36; *P* = 0.14, mean ± SEM). At that visit, standing height was measured using a Harpenden Stadiometer (London, UK). Weight was obtained using an electronic scale. Waist circumference (WC) was measured to the nearest 0.1 cm using a non-stretchable standard tape measure attached to a spring balance exerting a force of 750 gm. The measurement was taken over an unclothed abdomen at the smallest diameter between the costal margin and the iliac crest. Three measurements of height, weight and WC were taken and averaged. BMI (kg/m^2^) was calculated using average measurements of height and weight. Body composition was assessed using dual energy X-ray absorptiometry (DXA) on a GE Lunar Prodigy Advance (Model #8743) scanner. Blood lipid analyses and plasma insulin and glucose were analyzed by the McMaster Core Laboratory. Dysglycemia was classified according to the Canadian Diabetes Association guidelines and included prediabetes and diabetes. Prediabetes was defined as impaired fasting plasma glucose of 6.1–6.9 mmol/L or impaired glucose tolerance (2 h of 75 g OGTT) with a plasma glucose level between 7.8 to 11.0 mmol/L. Diabetes was defined as a fasting plasma glucose of ≥7.0 mmol/L and/or a 2 h plasma glucose during a 75 g OGTT of ≥11.1 mmol/L.

### Statistical analyses & Sample Size Calculation

All data were initially tested for normality using the D’Agostino and Pearson omnibus normality test. Normal data were analyzed using a student’s *t*–test or Pearson’s regression as indicated using GraphPad Prism 6.0. If the data were not normally distributed, they were log-transformed and re-tested for normality. Data that failed to conform to a normal distribution following transformation were analyzed using a Mann Whitney U test or Spearman regression. Proportions were compared using a Chi-square test. Multivariate analysis was performed using SPSS using dependent variables that were significantly associated in the univariate analysis and were either of interest (birth weight group) or have been previously associated with altered adipose tissue deposition (sex, body mass index). Significance was accepted as *P* < 0.05.

The measure of hepatic fat in young adults born prematurely has been done in one other small study^5^. Although our method for measuring hepatic fat differed, we utilized this study to calculate our sample size. With an *N* of 15 in each group we had >95% power to identify a difference in hepatic fat.

## Additional Information

**How to cite this article**: Crane, J. D. *et al.* ELBW survivors in early adulthood have higher hepatic, pancreatic and subcutaneous fat. *Sci. Rep.* **6**, 31560; doi: 10.1038/srep31560 (2016).

## Supplementary Material

Supplementary Information

## Figures and Tables

**Table 1:** Characteristics of the MRI study participants.

	NBW	ELBW	*P*-value
*n*	16	29	—
Sex (male/female)	7/9	12/17	0.58
Age (y)	34.9 ± 0.32	34.3 ± 0.33	0.22
Birth weight (kg)	3.33 ± 0.10	0.83 ± 0.02	<0.001*
Small for gestational age (*n*)	—	15	—
Height (m)	1.71 ± 0.03	1.64 ± 0.02	0.029*
Weight (kg)	72 ± 4.1	73.8 ± 2.8	0.74
BMI (kg/m^2^)	24.5 ± 1.0	27.4 ± 1.0	0.07
Overweight (25–29) by BMI (*n*)	5	10	0.82
Obese (≥30) by BMI (*n*)	1	9	0.056

Data are means ± SEM. *Significantly different (*P* < 0.05) from NBW group.

**Table 2:** Determinants of hepatic, pancreatic, subcutaneous and visceral fat in multivariate analysis.

	N	Unstd β	SEB	β	95% CI		*P*-value	Model		
					Lower bound	Upper bound		R^2^ adj	R^2^ change	*P*-value
**Hepatic fat fraction**	45							0.613	0.639	<0.001*
Birth weight group		−0.048	0.034	−0.138	−0.117	0.020	0.162			
Sex		−0.059	0.032	−0.175	−0.123	0.005	0.070			
BMI		0.024	0.003	0.732	0.017	0.030	<0.001*			
**Pancreatic fat fraction**	45							0.585	0.613	<0.001*
Birth weight group		−0.058	0.037	−0.158	−0.132	−0.017	0.124			
Sex		−0.119	0.035	−0.334	−0.188	−0.049	0.001*			
BMI		0.022	0.003	0.649	0.015	0.029	<0.001*			
**Subcutaneous fat area**	44							0.641	0.666	<0.001*
Birth weight group		−0.038	0.055	−0.065	−0.148	0.073	0.494			
Sex		0.162	0.052	0.288	0.058	0.266	0.003*			
BMI		0.043	0.005	0.761	0.032	0.054	<0.001*			
**Visceral fat area**	44							0.711	0.731	<0.001*
Birth weight group		−0.003	0.049	−0.005	−0.101	0.096	0.952			
Sex		−0.191	0.046	−0.341	−0.284	−0.098	<0.001*			
BMI		0.043	0.005	0.765	0.033	0.053	<0.001*			

β, standardized regression coefficient.
