# Supplementary material for: ELBW survivors in early adulthood have higher hepatic, pancreatic and subcutaneous fat
Source: Sci Rep. 2016 Aug 17;6:31560. doi: 10.1038/srep31560 (PMC4987614; doi:10.1038/srep31560)
Supplement: Supplementary Information [file srep31560-s1.pdf]

Supplementary Information

ELBW survivors in early adulthood have higher hepatic, pancreatic and subcutaneous fat

Justin D. Crane, Samuel A. Yellin, Frank J. Ong, Nina P. Singh, Norman Konyer, Michael D. Noseworthy, Louis A. Schmidt, Saroj Saigal and Katherine M. Morrison

Supplementary Table S1. Univariate regression analyses of organ fat

	Liver fat (%)	Pancreatic fat (%)	Subcutaneous fat (cm^2)	Visceral fat (cm^2)
Liver fat	–	**0.721**	**0.556**	**0.740**
Pancreatic fat	–	–	**0.609**	**0.871**
Subcutaneous fat	–	–	–	**0.652**
Visceral fat	–	–	–	–

Data are r-values performed using Pearson or Spearman correlations. Significant data (*P* < 0.05) are indicated in bold.

Supplementary Table S2. Prior characteristics of the study participants collected 2 years prior to the MRI data.

	**NBW**	**ELBW**	***P*-value**
Height (m)	1.71 ± 0.03	1.64 ± 0.02	**0.026***
Weight (kg)	74 ± 3.9	71.6 ± 2.6	0.56
BMI (kg/m^2)	25.3 ± 0.9	26.8 ± 1.1	0.34
Overweight (25-29) by BMI (*n*)	7	8	0.33
Obese (≥30) by BMI (*n*)	1	8	0.13
WC (cm)	83.3 ± 3.0	83.2 ± 2.3	0.99
WC/height (cm/cm)	0.49 ± 0.02	0.51 ± 0.02	0.33
LMI (kg/m^2)	16.6 ± 0.7	16.3 ± 0.5	0.70
Body fat (%)	30.5 ± 2.2	34.3 ± 2.3	0.29
Systolic BP (mmHg)	108 ± 3	112 ± 2	0.26
Diastolic BP (mmHg)	70 ± 2	72 ± 2	0.56
Total cholesterol (mmol/L)	4.7 ± 0.2	4.6 ± 0.2	0.88
LDL cholesterol (mmol/L)	2.9 ± 0.2	2.6 ± 0.2	0.32
HDL cholesterol (mmol/L)	1.4 ± 0.1	1.5 ± 0.1	0.29
Triglyceride (mmol/L)	1.1 ± 0.2	1.3 ± 0.1	0.26
Fasting blood glucose (mmol/L)	5.0 ± 0.1	5.2 ± 0.1	0.67
OGTT: 120 min blood glucose (mmol/L)	5.1 ± 0.4	6.2 ± 0.4	0.10
Fasting insulin (pmol/L)	66.2 ± 8.9	68.2 ± 6.0	0.82
OGTT: 120 min insulin (pmol/L)	271.3 ± 49.5	442.1 ± 74.49	0.07
HOMA-IR	1.23 ± 0.16	1.28 ± 0.11	0.80
Impaired fasting glucose (*n*)	0	0	–
Impaired glucose tolerance (*n*)	1	3	0.57
Impaired fasting glucose + impaired glucose tolerance (*n*)	1	1	0.72
Diabetic (*n*)	0	3	0.16
Hypertensive (*n*)	0	1	0.46

Data are means±SEM. *Significantly different ($P < 0.05$) from NBW group and indicated in bold. WC, waist circumference; LMI, lean mass index; BP, blood pressure. $n = 26$ for ELBW 2hr OGTT plasma glucose and $n = 28$ for fasting plasma glucose.
